# CD4 + CD25 + CD127 high cells as a negative predictor of multiple organ failure in acute pancreatitis

**DOI:** 10.1186/s13017-017-0116-7

**Published:** 2017-02-02

**Authors:** Wei Wang, He-Ping Xiang, Hui-Ping Wang, Li-Xin Zhu, Xiao-Ping Geng

**Affiliations:** 1 Department of Emergency Surgery, The Second Affiliated Hospital of Anhui Medical University, 678 Furong Road, Hefei, 230601 Anhui Province People’s Republic of China; 2 Hematology department, The Second Affiliated Hospital of Anhui Medical University, Hefei, 230601 Anhui Province People’s Republic of China; 3 Central lab of the First Affiliated Hospital of Anhui Medical University, Hefei, 230022 Anhui Province People’s Republic of China; 4 Department of General Surgery, The Second Affiliated Hospital of Anhui Medical University, Hefei, 230601 Anhui Province People’s Republic of China

**Keywords:** Acute pancreatitis, Prognosis, Multiple organ failure, CD4 + CD25 + CD127^high^ cell, Regulatory T cell, Natural killer cells

## Abstract

**Background:**

It has been suggested that severity of the immune response induced by immune cells is associated with morbidity and mortality from acute pancreatitis. The authors investigated and evaluated the relationship between distinct peripheral lymphocyte subsets at admission and clinical outcome prior to hospital discharge so as to find a predictor to the prognosis of acute pancreatitis in lymphocyte profile.

**Methods:**

Lymphocyte subsets in admission peripheral venous blood were tested through flow cytometry on 48 patients with acute pancreatitis. Clinical data was recorded as well. The primary observational outcomes were multiple organ failure (MOF) and infection.

**Results:**

There was a significant difference in natural killer cells between two subgroups sorted by the presence or absence of infection (25.5 ± 4.47 [95% CI 14.4, 36.6] *vs* 14.8 ± 7.62 [95% CI 12.5,1 7.1] *p* = 0.021). Patients who developed MOF had lower CD4 + CD25 + CD127^high^ (4.49 ± 1.5 (MOF) [95% CI 3.83, 5.16] *vs* 6.57 ± 2.65 (non-MOF) [95% CI 5.5, 7.64] *p* = 0.002) and higher CD127low/high cell counts (1.35 ± 0.66 [95% CI 1.06, 1.65] *vs* 0.97 ± 0.44 [95% CI 0.79, 1.15] *p* = 0.02). MOF patients were significantly older (55 ± 14.58 [95% CI 48.49,61.42] *vs* 46 ± 15.59 [95% CI 39.39,51.99] *p* = 0.04), and had higher Acute Physiology and Chronic Health Evaluation IIscores (7 ± 3.66 [95% CI 5.5,7.64] *vs* 4 ± 2.89 [95% CI 2.45,4.78] *p* = 0.001) and C reactive protein (100.53 ± 94.38 [95% CI 58.69,142.48] *vs* 50.8 ± 59.2 [95% CI 26.88,74.71] *p* = 0.04). In a multivariate regression model, only CD4 + CD25 + CD127^high^ cell was a significant predictor of non-MOF. For the detection of non-MOF, CD4 + CD25 + CD127^high^ cell generated a receiver operating characteristic (ROC) curve with an area under the curve of 0.74.

**Conclusion:**

CD4 + CD25 + CD127^high^ cell at early phase of acute pancreatitis yields good specificity in detecting non-MOF at a suggested cutoff value 6.41%. Patients with fewer natural killer cells may be at risk in developing secondary infection.

## Background

Acute pancreatitis (AP) is an inflammatory disorder associated with high morbidity and mortality rate. The annual incidence of acute pancreatitis ranges from 13 to 45 per 100 000 people [1]. AP is the leading discharge diagnosis in patients admitted with gastrointestinal or liver problems in countries such as the United States [2]. Prognosis of the disease varies widely, from self-limiting to severe or critical, and mortality reaches 30% in severe cases [3].

Irrespective of the etiology of pancreatitis, early pathophysiology events include activation of digestive enzymes by lysosomal hydrolases, autodigestive processes, intra-parenchymal inflammatory response and the systemic inflammatory response syndrome (SIRS). The initial protease cascade doesn’t necessarily determine the severity of AP [4]. In contrast, evidence is accumulating of a crucial relationship between innate immune components involved in AP pathogenesis and disease severity [5–7]. Neutrophils and macrophages are the first line of the immune system’s defense. T-lymphocytes, which are mainly involved in the cell-mediated immune response, also play a vital role in AP [5]. CD4 + T cells increase the severity of AP due to macrophage activation via antigen presentation and pro-inflammatory cytokine release as well as through direct cytotoxicity effects [5]. Immunological suppression mediated by regulatory T cells (Tregs) expressing transcription factor forkhead box P3 (FOXP3) has been reported to be the critical mechanism controlling the inflammatory response for which the immune system is primed after serious injury such as severe AP (SAP) [8]. However, these studies focused on a single lymphocyte subgroup rather than the broad subgroups of lymphocyte in patients with AP.

In present study, we evaluated the relationship between multiple peripheral lymphocyte subsets (i.e., T lymphocyte cells, T Helper cells, cytotoxic T cells, Tregs, activated effector T cells, natural killer (NK) cells and B cells) through flow cytometry done early during hospitalization vs. patient outcome. We then compared the accuracy of activated effector T cells estimation to predict non-MOF with Acute Physiology and Chronic Health Evaluation (APACHE) IIscores and C reactive protein (CRP) estimation of MOF.

## Methods

This prospective clinical observational study was performed between June 2015 and August 2015 in the second affiliated hospital of Anhui Medicine University. The study was approved by the hospital’s Ethics Committee. Written informed consent was obtained from patient surrogates before study inclusion.

### Eligible criteria and treatment

Patients who achieved two of the following three features were diagnosed as having AP and screened for participation in the study: acute epigastric pain with or without radiation through to the back; serum amylase or lipase activity greater than three times the upper limit of normal; and characteristic features on cross-sectional abdominal computer tomography (CT) imaging consistent with the diagnosis of acute pancreatitis [9].Exclusion criteria were: age <18 or >80 years, previous or chronic treatment with immunological suppressants such as cortisone or its analogues, immunodeficiency diseases such as acquired immune deficiency syndrome (AIDS), malignant tumor, ongoing radiotherapy or chemotherapy, pregnancy, or patients without written permission or complete data. Of the 60 cases identified, 48 patients were eligible for inclusion in the study. Among 12 patients who were excluded from the study, 8 patients exceeded the study age limit, 2 patients were pregnant, 1 patient persisted in taking glucocorticoid due to rheumatoid arthritis, and the last one was undergoing chemotherapy after radical gastrectomy.

**Fig. 1:**
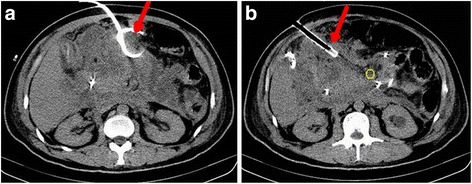
A 28-year-old man with acute necrotizing pancreatitis complicated by infected pancreatic necrosis requiring multiple percutaneous catheter drainage (red arrow in A and B). There is a large heterogeneous area of necrosis in the pancreatic and peri-pancreatic area with impacted gas bubbles (yellow ring in B)

Eligible patients were included as soon as possible at time of hospital admission. All patients were treated according to standard guidelines including fluid resuscitation, enteral nutrition, endoscopic retrograde cholangio-pancreatography (ERCP), catheter drainage (Fig. 1) and necrosectomy when indicated [10].

### Sample collection and preparation

Each patient’s peripheral venous blood (2 ml) was collected in an anticoagulation tube containing heparin within two hours of admission. Heparin (100ul) was added to each blood sample in 2 tubes separately. Next, 10ul of mixed antibodies was added to one of the tubes. A second control tube was mixed with 10ul Isotype Control Antibodies. The tubes were mixed gently and incubated for 15 min in the dark. A hemolytic agent (0.83% NH_4_Cl) was put in 2 tubes and incubated for 10 mins in a 37 °C water bath.

### Flow cytometry analysis

**Fig. 2:**
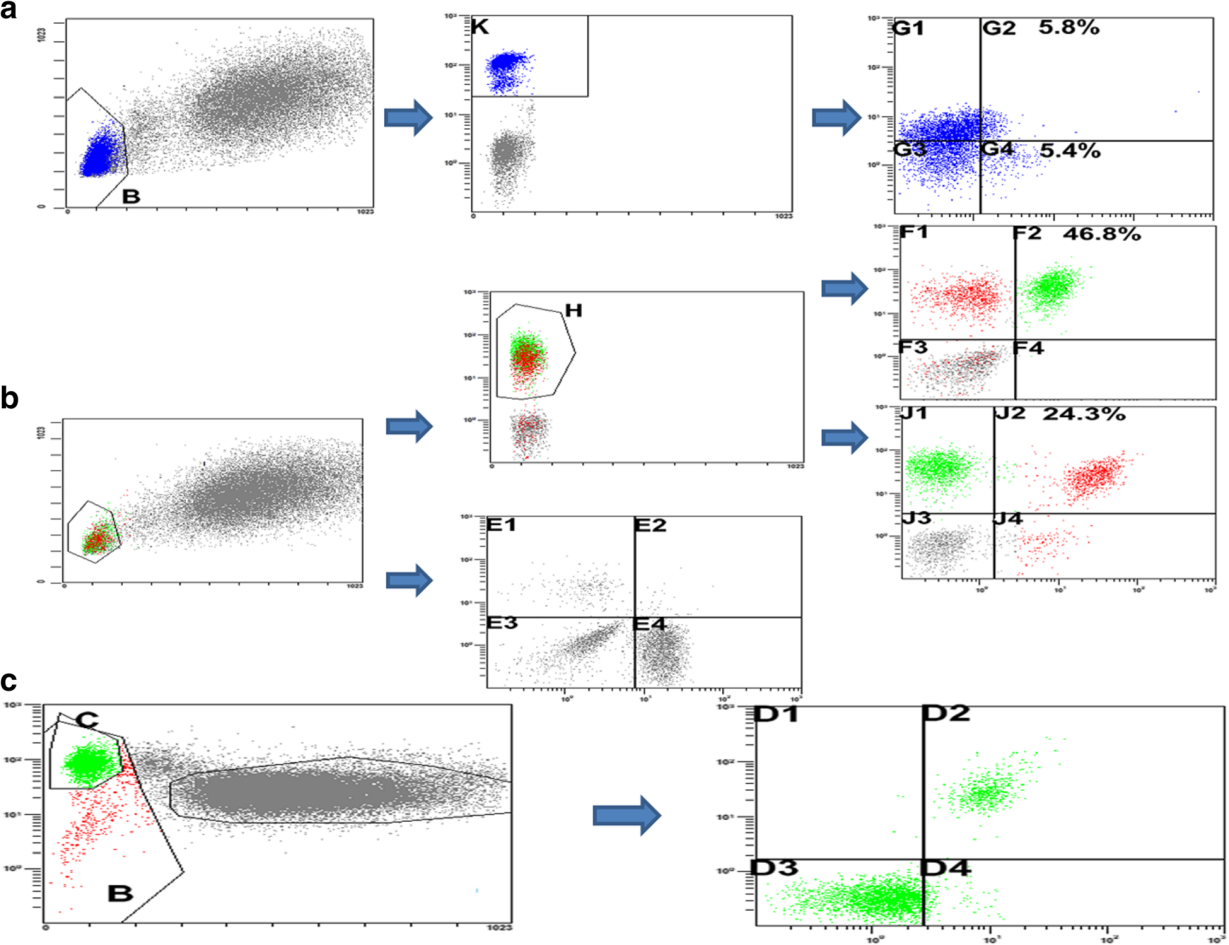
Gating strategy of peripheral lymphocyte population (**a**) Gating strategy for CD4+ T cell subdivided into CD4 + CD25 + CD127^low^ and CD4 + CD25 + CD127^high^ subpopulation. B indicate lymphocyte divided from leukocyte based on forward scatter and side scatter, K indicate CD4+ T cells, G2 indicate CD4 + CD25 + CD127^high^ cells and G4 CD4 + CD25 + CD127^low^ cells. **b** Gating strategy for lymphocyte subdivided into T Helper cells, cytotoxic T cells and NK cells subpopulation. H indicate CD3+ T cells, F2 indicate CD3 + CD4+ T cells and J2 CD3 + CD8+ T cells, E1 indicate CD3-CD16 + CD56+ cells which were divided from CD3- T cells. **c** Gating strategy for B cells. C indicate lymphocyte divided from leukocyte according to CD45 and side scatter. D2 indicate CD19 + CD20 + CD45+ cells

Lymphocyte subsets in blood were phenotyped by flow cytometry (Beckman-Coulter, Brea, CA) according to the manufacturer’s instructions. We first gated on lymphocytes based on forward scatter and side scatter; >10,000 cells within gate were obtained for every sample. Each antibody was matched with a respective isotype IgG1 as a control, and the gating threshold was set accordingly. Cells were labeled with specific mono-antibodies in different combinations. Lymphocyte subsets were selected for detailed phenotypic analysis as follows: T lymphocyte cells were CD3+ T cells; T Helper cells were CD3 + CD4+ T cells; cytotoxic T cells were CD3 + CD8+ T cells; NK cells were CD3-CD16 + CD56+ T cells; B cells were CD19 + CD20 + CD45+ T cells; Tregs were CD4 + CD25 + CD127^low^ T cells; and activated effector T cells were CD4 + CD25 + CD127^high^ T cells. Representative gating figures are shown in Fig. 2.

### Endpoints and outcome assessment

The primary observational endpoints of the study were MOF and infection. Severity of AP was divided into three categories: mild, moderate severity (MSAP), and severe (SAP) according to the new revised Atlanta consensus. Organ failure was defined as a Sepsis-related Organ Failure Assessment (SOFA) score ≥2. MOF was defined as failure of two or more organs [11]. Infection was diagnosed based on ongoing signs of sepsis and/or the combination of clinical signs and CT imaging when extra-luminal gas was present within areas of necrosis in the pancreatic and/or peri-pancreatic tissues (Fig. 1). Balthazar scores were used to assess the extent of local inflammatory changes [1]. Second study endpoints were mortality before discharge and intensive care unit (ICU) length of stay (LOS).

We recorded demographic data, comorbid, clinical laboratory values, treatment procedure and outcome and calculated the APACHE II score, SOFA score and Balthazar score in the first 24 h after admission. All data were entered into a secure and pre-established case report form by trained research assistants.

### Statistical analysis

The study population was characterized using descriptive statistics. Categorical variables are provided as counts with frequencies and continuous variables as means with standard deviations (SD) or medians with quartiles depending on the normality of the data. Kolmgorov-Smirnov test was used to test the normal distribution of the data.

Group comparisons used the Student *t*-test for continuous variables, Chi-square for categorical data, and the ANOVA test and Mann–Whitney test for ordinal data. Spearman correlation analysis was used to determine the degree of relationship between lymphocytes substrate and other laboratory values and scores. Binary logistic regression was used for multivariate analysis. Only variables which were significantly associated with primary outcomes (including MOF and infection in univariate analysis) were introduced into the multivariate logistic model. Receiving operator characteristic (ROC) curves were generated with corresponding area under curve (AUC) analysis and computation of 95% confidence intervals (CI). The AUC was calculated as a measure of the ability for each marker to distinguish between groups. The optimum predictor cut-off was calculated as a trade-off between sensitivity and specificity. All hypothesis tests were two-sided, with a significance level of *p* < 0.05. Statistical analyses were performed using SPSS version 19.0 (IBM, Chicago, IL).

## Results

### Patient characteristics

The study included 48 patients. No patients were lost to follow up and no included patients had incomplete clinical data. The mean age for the complete study population was 50 (SD: 16) years and 26 (54%) were male. Gallstones were the major cause of AP with an incidence of 39.6%. Baseline patient characteristics including laboratory tests at admission are presented in Table 1. Three (6.25%) patients developed infection and presented with sepsis and pancreatic abscess (Fig. 1). All recovered fully after receiving appropriate antibiotics and multiple percutaneous, CT-guided external drainages (Fig. 1). All abscess pathogens were confirmed Gram-negative bacteria including Escherichia coli and Enterococcus faecium. Twenty-two patients (45.8%) developed subsequent MOF. One of these patients (2%) died before hospital discharge. Median hospital LOS was 10 days.

**Table 1:** Selected baseline characters of the study patients^abc^

Age(years)	50 ± 16
Male sex	26(54.17%)
Etiology	
gallstone	19(39.6%)
hypertriglyceride	15(31.2%)
alcohol	4(8.3%)
idiopathic	10(20.9%)
Category	
MAP	21(43.8%)
MSAP	19(39.6%)
SAP	8(16.6%)
Amylase(IU) at admission	720(349.5–1581.25)
CRP(mg/L) at admission	73.59 ± 80.44
Comorbidities	17(35.42%)
APACHEIIscores at enrollment	5.19 ± 3.66
SOFA scores at enrollment	2(1–3)
Balthazar scores at enrollment	2(2–3.75)
Lymphocyte (%)	16.24 ± 9.60
Lymphocyte subsets	
B cell (%)	7.84 ± 5.89
T cell (%)	68.92 ± 9.69
Helper T cell (%)	42.65 ± 11.31
Cytotoxic T cell (%)	23.38 ± 7.46
NK cell (%)	15.49 ± 7.88
Tregs (%)	5.55 ± 1.69
Activated effector T cell (%)	5.62 ± 2.42
CD4+/CD8+	2.13 ± 1.18
CD127low/high	1.15 ± 0.58

^a^Categorical variables are presented as count (frequency) and continues variables as mean (standard deviation) or median (quartiles) depending on the normality of the data

^b^CRP: C-reactive protein, APACHE: Acute Physiology and Chronic Health Evaluation, Tregs: T regular cell

^c^the value of the lymphocyte subsets including B cell, T cell, Helper T cell, Cytotoxic T cell, NK cell is the percentage to total lymphocytes. Whereas the value of two subsets including Treg cell and activated effector T cell is the percentage to helper T cell

### Univariate analysis

**Fig. 3:**
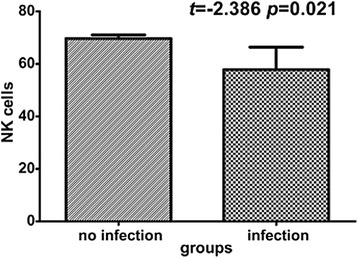
mean NK cells percentage of lymphocytes at admission in infection group vs non- infection

Patients were stratified to two subgroups based on the presence or absence of MOF and infection, and the clinical variables were compared between subgroups. Figure 3 shows that NK cells increased significantly in the infection subgroup (*p* = 0.021). Activated effector T cells were significantly decreased in the MOF subgroup (*p* = 0.002) while the ratio of CD4 + CD25 + CD127^low^ and CD4 + CD25 + CD127^high^ subsets (CD127low/CD127high) was significantly increased in the MOF subgroup (*p* = 0.02). There was no difference in other lymphocytes subsets between subgroups. Patients in the MOF subgroup were older (*p* = 0.04) and had higher APACHEIIscores (*p* = 0.001) and CRP (*p* = 0.04) (Table 2). There were no associations between LOS and either MOF (rho = 0.084 *p* = 0.573) or infection (rho = 0.213 *p* = 0.147).

**Table 2:** Univariate analysis of selected variables between subgroups sorting with MOF

	MOF (*n* = 22)	Non-MOF (*n* = 26)	*P* value
Age(years)	55 ± 14.58	46 ± 15.59	0.04
APACHEIIscores	7 ± 3.66	4 ± 2.89	0.001
CRP(mg/L)	100.53 ± 94.38	50.8 ± 59.2	0.04
Tregs (%)	5.51 ± 1.87	5.59 ± 1.56	0.879
Activated effector T cell (%)	4.49 ± 1.5	6.57 ± 2.65	0.002
CD4+/CD8+	2.2 ± 1.34	2.07 ± 1.04	0.71
CD127low/high	1.35 ± 0.66	0.97 ± 0.44	0.02

*CRP* C-reactive protein, *APACHE* Acute Physiology and Chronic Health Evaluation, *Tregs* T regular cell, *MOF* multiple organ failure

### Multivariate analysis for MOF

Multivariate logistic regression was performed adjusting for the effects of potentially confounding variables to predict MOF as a function of activated effector T cells, CD127low/high, and age. These variables were selected by investigating the association of all variables in Table 2 with MOF and selecting variables with significant associations as potential confounders. Using this multivariate regression model, only the variable, activated effector T cells (OR = 0.564 *p* = 0.042 95% CI [0.324, 0.98]), was a significant negative predictor of MOF (Table 3).

**Table 3:** Multiple logistic regression model for MOF

predictor	Frequency of MOF		
	OR	95%CI	*P* value
age	1.044	0.996–1.094	0.073
Activated effector T cell (%)	0.564	0.324–0.98	0.042
CD127low/high	0.881	0.156–4.977	0.886

*MOF* multiple organ failure

### ROC Curve of activated effector T cells in Predicting non-MOF and APACHEIIscores and CRP in predicting MOF

**Fig. 4:**
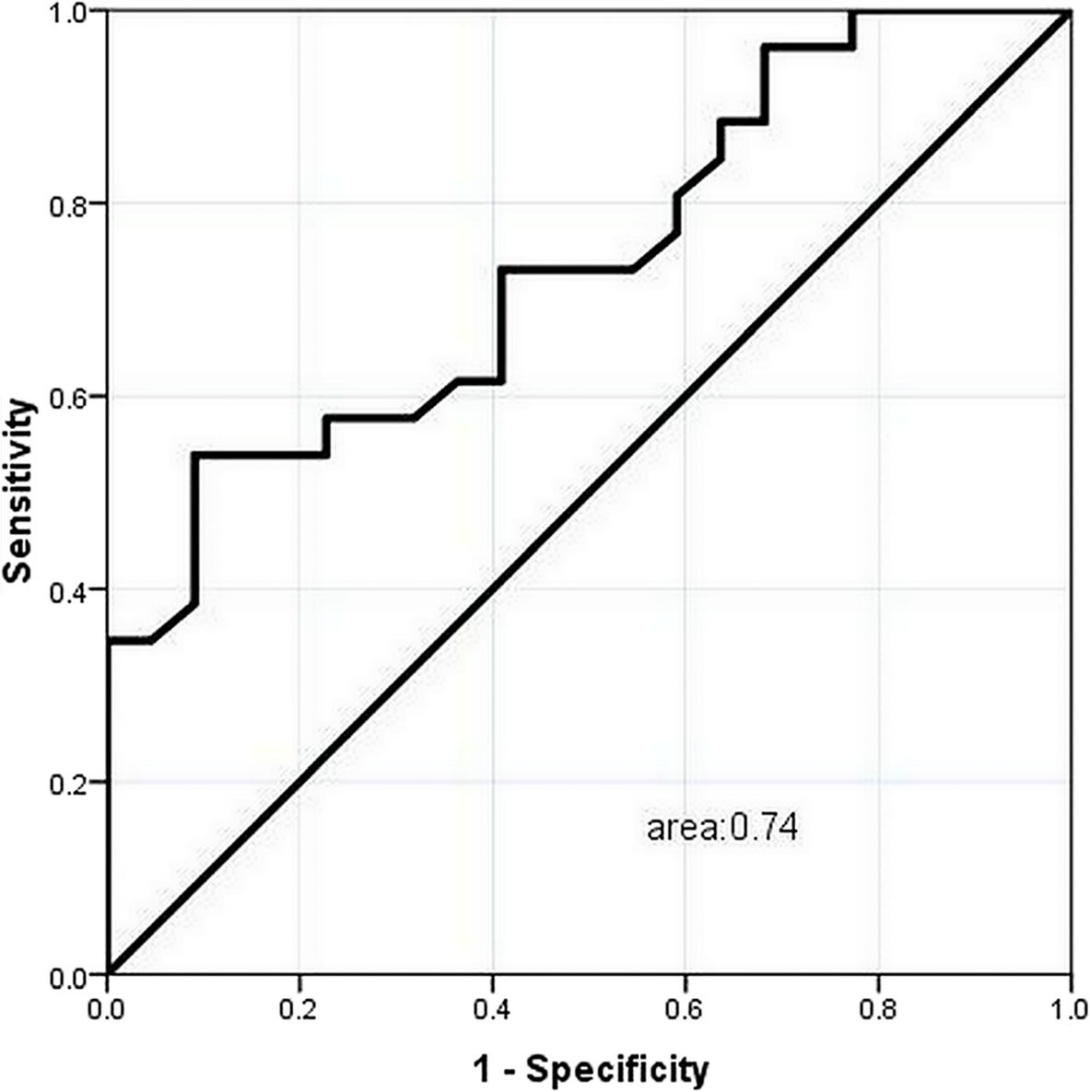
ROC curve of CD4 + CD25 + CD127^high^ cell in predicting non-MOF developing in the progress of acute pancreatitis

**Fig. 5:**
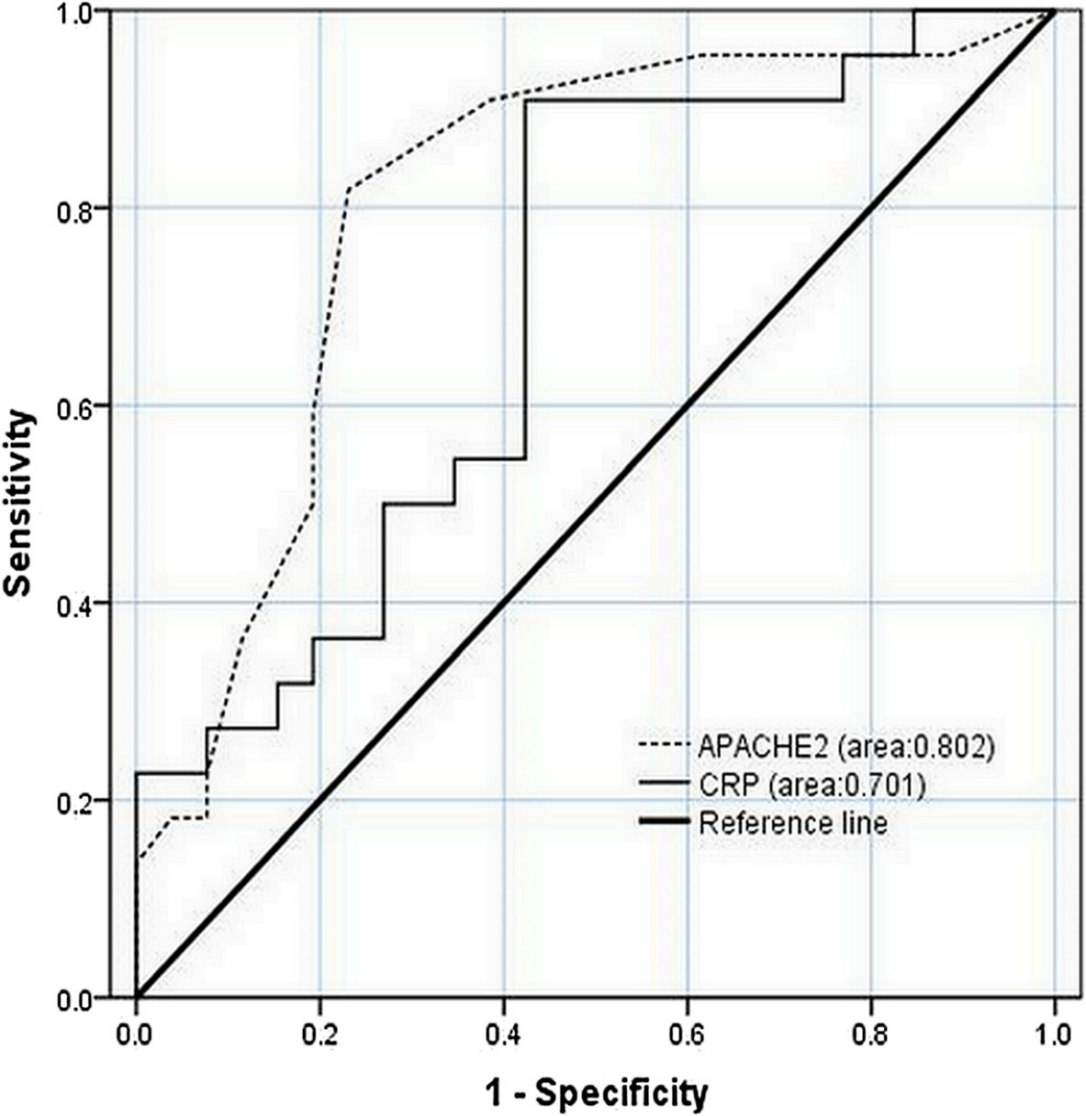
ROC curve of APACHEIIversus CRP in predicting MOF developing in the progress of acute pancreatitis

Figure 4 illustrates the superiority of activated effector T cells (area under the curve 0.74) in predicting non-MOF. Activated effector T cells at 6.41% could be used as a cut-off point to predict non-MOF with a sensitivity of 53.8%, specificity of 90.9%. In contrast, both APACHEIIand CRP exhibited powerful ability to predict MOF with areas under the curves of 0.802 and 0.701, respectively, indicating that the former was superior than the latter based on the ROC curves (Fig. 5).

## Discussion

The principle findings of our study are: (1) among peripheral lymphocyte subsets, only activated effector T cells phenotyped by CD4 + CD25 + CD127^high^ have a significant negative correlation with MOF and can be used as a predictor of non-MOF estimation in AP; and (2) there is a statistically significant association between NK cells at admission and secondary infection.

AP can present as a severe acute abdominal disease with a high mortality. Patients with AP can develop an early-onset phase of SIRS within 2 weeks, and/or an infection phase due to pancreatic necrosis. MOF and/or infection signal the presence of severe disease, and there is a high risk of death if both are present [11]. Thus, both MOF and infection are unfavorable prognostic signs in AP patients.

In the present study, we did not find correlations between activated effector T cells and both SOFA scores and Balthazar scores (*r* = −0.276 *p* = 0.058 and *r* = −0.140 *p* = 0.342 respectively). In contrast, there were correlations between activated effector T cells and both APACHE IIscores and CRP. Furthermore, there were significant differences in the level of activated effector T cells among three subgroups stratified by Revision of Atlanta criteria (*F* = 5.26 *p* = 0.009), and the severe AP patients had the lowest level of activated effector T cells (5.36% ± 1.31%). From the result, it was demonstrated that activated effector T cells enrollment was in accordance with Ranking criteria, APACHE IIscores and CRP rather than Balthazar scores and SOFA scores. Balthazar scores based on the CT findings focus on local complications of AP and it is hard to reflect the systematic immunology condition. In terms of SOFA scores, it is not a normal clinical predictor in assessing severity of AP. considering the potential confounding bias caused by accumulative SOFA scores, it was just utilized for judging whether it was MOF individually in the present study.

An accurate predictor to prognosis would be helpful for clinicians by allowing patients with a predicted severe course to be transferred to an intensive care unit while patients with a predicted mild course may be treated in an outpatient setting. Severity scoring systems such as APACHEIIand laboratory testing such as CRP have been used to accurately predict an unfavorable outcome, which we have confirmed in the present study [12, 13]. However, there has not been a validated, published strategy to predict a favorable outcome, which is important to avoid overtreatment. In the present study, only a special T cell subset called CD4 + CD25 + CD127^high^ cells using the cutoff value >6.41% at admission was a statistically significant negative predictor of AP severity upon patient presentation to the hospital. Even though this predictor had a low sensitivity of 53.8%, we believe that the specificity of such a value is of more clinical importance than its sensitivity because conservative and supportive management is the initial care for patients with AP and it seems unnecessary to apply drastic remedies for those with higher CD4 + CD25 + CD127^high^ cells. This test’s potential as a superior predictor of a favorable prognosis will require confirmation in a larger population.

Lymphocytes play important roles in both adaptive and innate immune responses. The main function of the lymphocytic immune response is to mediate and resolve the nonspecific inflammatory process in AP. CD4+ T cells may act as co-stimulators for macrophage activation via antigen presentation and pro-inflammatory cytokine release as well as through direct cytotoxic effects (on acini) through Fas ligand expression on CD4+ T cells [14]. In addition, a distinct set of CD4 + CD25 + FOXP3 + Tregs presenting immunological tolerance and homeostasis have been identified with anti-inflammatory functions in AP [15]. These cells could bind to multiple effector immune cells and prevent their secretion of cytokines. They also secrete anti-inflammatory cytokines such as interleukin (IL)-10 and transforming growth factor (TGF)-β. Tregs have also been reported to restrain SIRS [16]. It was impossible to purify living Tregs in vivo previously because of the exclusive intracellular expression of FOXP3 as a critical factor for Tregs function. Recently, CD127 (IL-7 receptor a chain), whose expression was reverse to FOXP3, was recommended as an marker of Treg and has confirmed superiority compared to other cell surface markers [17, 18]. As indicated in previous study, serum sCD163, a biomarker released from macrophages, was increased in AP, but not associate with disease severity [19]. In present study, we used the sorting strategy of combining CD4+, CD25+, and CD127low to obtain viable, expandable Treg cells. There was no significant difference in Tregs between the two groups statistically, and we also found the mean number of Tregs as a percentage of CD4+ T cells was 5.55 ± 1.69%, which was a little lower than the 6.25 ± 0.26% in healthy people reported in previous research [18].

Prior studies have come to different conclusions regarding variation of Tregs in SIRS or the early phase of AP. Xue H et al. [20] reported that CD4 + CD25 + Tregs increased in SIRS and suppressed the excessive inflammatory response. In contrast, other investigators have reported that positive CD4 + CD25+ T cells significantly declined in a mouse SAP model [8]. Data on Tregs in humans with AP is limited and our data may be of value as a reference for future study.

Interestingly, the novel subgroup CD4 + CD25 + CD127^high^ cells had predictive value for the development of non-MOF in this investigation. This subset was previously thought to be a contaminant interfering with the suppressive function of Tregs and was neglected previously.[18] However, research by Michel L indicates that this specific population in multiple sclerosis (MS) appears more proliferative and secretes more interferon-γ (IFN-γ) and interleukin-2 (IL-2), both pro-inflammatory cytokines, than healthy individuals [21]. Similar observation has been made in CD4 + CD25 + CD127^high^ cells infiltrating rejected human allografts, in which allo-specific CD4 + CD25 + CD127^high^ cells were able to secrete effector cytokines such as tumor necrosis factor-α (TNF-α) and IFN-γ [22]. The ratio of CD127low/high also was statistically different in subgroups with and without MOF, but had no correlation with MOF in our multivariate regression model. Thus, CD4 + CD25 + CD127^high^ cells counts may be the sole factor which are associated with MOF estimation, and it is postulated that increment of CD4 + CD25 + CD127^high^ cells have a protective function to reflect the progress of AP independent of Tregs. The exact mechanism of CD4 + CD25 + CD127^high^ cell function in the setting of AP needs further study.

NK cells with phenotype marker CD3-CD16 + CD56+ are the type of cytotoxic lymphocytes critical to the innate immune system and secrete TNF-α and IFN-γ to control viral infection [23]. Dabrowski A et al. [24] found a dramatic depletion of peripheral NK cells in SAP compared to MAP and control, which indicates that there may be a correlation between NK cells and severity of AP. Our research further found that a lower number of NK cells at admission were associated with the development of secondary infection in AP patients. Since most pathogens causing secondary pancreatic infection are Gram-negative bacteria such as Escherichia coli originating from gut [25], we hypothesize that once circulating NK cells run into Gram-negative bacteria in a primary infection, they acquire memory function features which are a hallmark of T and B cells belonging to the adaptive immune system [26]. The memory-like, circulating NK cells then migrate into the pancreas at the early onset of AP and trigger a rapid immune response to the same antigen encountered again. In any case, patients with lower NK cells at admission may have a high risk of developing secondary infection in AP.

### Limitations

Specific limitations need to be considered when interpreting results of our study. First, the number of study patients included (*n* = 48) and infection group (*n* = 3) was low. Furthermore, sepsis in the secondary progression of AP also triggers MOF which is more likely to happen due to SIRS in the first phase. The selection bias without intent may affect extrapolation of our outcomes, especially regarding the predictor role of activated effector T cells to non-MOF. Nonetheless, statistical significance was achieved for several analyses suggesting that these relationships could be clinically relevant. Second, the observational nature of this study indicated that relationships between dependent and independent variables are associations, and not cause and effect. Third, a separate test might be needed in different phases of the disease because of the essential dynamic progress of immune function during the development of AP. The present study did not dynamically investigate the immune function. Therefore, it can’t reflect comprehensive immunological fluctuation.

## Conclusion

Increased peripheral CD4 + CD25 + CD127^high^ cells, whose physiological role in the early phase of AP is unclear in the early phase of AP, appear to be associated with a good (non-MOF) prognosis. In addition, patients with lower NK cells at admission may have a higher risk of developing secondary infection in AP. Further study is needed to confirm these observations.

## Abbreviations

AIDS: Acquired immune deficiency syndrome
AP: Acute pancreatitis
APACHE: Acute Physiology and Chronic Health Evaluation
CD: Cluster of differentiation
CRP: C reactive protein
CT: Computer tomography
FOXP3: Forkhead box P3
ICU: Intensive care unit
IFN-γ: Interferon-γ
IL: Interleukin
LOS: Length of stay
MOF: Multiple organ failure
NK: Natural killer
ROC: Receiver operating characteristic
SIRS: Systemic inflammatory response syndrome
SOFA: Sepsis-related Organ Failure Assessment
TGF: Transforming growth factor
TNF: Tumor necrosis factor.
Tregs: Regulatory T cells
